# Human and animal exposure to newly discovered sand fly viruses, China

**DOI:** 10.3389/fcimb.2023.1291937

**Published:** 2024-01-03

**Authors:** Xiaohui Yao, Qikai Yin, Xiaodong Tian, Yuke Zheng, Hongyan Li, Shihong Fu, Zhengmin Lian, Yijia Zhang, Fan Li, Weijia Zhang, Ying He, Ruichen Wang, Bin Wu, Kai Nie, Songtao Xu, Jingxia Cheng, Xiangdong Li, Huanyu Wang, Guodong Liang

**Affiliations:** ^1^ Department of Arbovirus, National Key Laboratory of Intelligent Tracking and Forecasting for Infectious Diseases, National Institute for Viral Disease Control and Prevention, Chinese Center for Disease Control and Prevention, Beijing, China; ^2^ Jiangsu Co-innovation Center for Prevention and Control of Important Animal Infectious Diseases and Zoonoses, College of Veterinary Medicine, Yangzhou University, Yangzhou, China; ^3^ Department of Vector Biology, Shanxi Province Center for Disease Control and Prevention, Taiyuan, Shanxi, China; ^4^ Yangquan Center for Disease Control and Prevention, Yangquan, Shanxi, China

**Keywords:** China, Hedi virus, Wuxiang virus, *Phenuiviridae*, seroprevalence, infection of sand fly viruses, zoonotic pathogens

## Abstract

**Introduction:**

The Hedi virus (HEDV) and Wuxiang virus (WUXV) are newly discovered Bunyaviruses transmitted by sandflies. The geographical distribution of isolation of these two viruses continues to expand and it has been reported that WUXV causes neurological symptoms and even death in suckling mice. However, little is known about the prevalence of the two viruses in mammalian infections.

**Methods:**

In order to understand the infection status of HEDV and WUXV in humans and animals from regions where the viruses have been isolated, this study used Western blotting to detect the positive rates of HEDV and WUXV IgG antibodies in serum samples from febrile patients, dogs, and chickens in the forementioned regions.

**Results:**

The results showed that of the 29 human serum samples, 17.24% (5/29) tested positive for HEDV, while 68.96% (20/29) were positive for WUXV. In the 31 dog serum samples, 87.10% (27/31) were positive for HEDV and 70.97% (22/31) were positive for WUXV, while in the 36 chicken serum samples, 47.22% (17/36) were positive for HEDV, and 52.78% (19/36) were positive for WUXV.

**Discussion:**

These findings suggest there are widespread infections of HEDV and WUXV in mammals (dogs, chickens) and humans from the regions where these viruses have been isolated. Moreover, the positive rate of HEDV infections was higher in local animals compared to that measured in human specimens. This is the first seroepidemiological study of these two sandfly-transmitted viruses. The findings of the study have practical implications for vector-borne viral infections and related zoonotic infections in China, as well as providing an important reference for studies on the relationship between sandfly-transmitted viruses and zoonotic infections outside of China.

## 1 Introduction

HEDV (Hedi virus) and WUXV (Wuxiang virus) are viruses that have been isolated from sandflies collected from the natural environment in recent years. Molecular genetic analyses of these two viruses indicate that they belong to a newly identified genus of *Phlebovirus*, which falls within the *Bunyavirales* order and the *Phenuiviridae* family (Wang et al., 2020; Calisher and Calzolari, 2021; Xu et al., 2021; Wang et al., 2022). Similar to other *Bunyavirales*, HEDV and WUXV are enveloped, negative-sense RNA viruses with a spherical morphology, ranging in diameter from 80 to 120 nm. Their genomes consist of three segments, S, M, and L (Ter Horst et al., 2019; Wang et al., 2021). The S segment encodes the nucleoprotein (NP) and the nonstructural protein (NSP), NP, which is a critical component of both viral ribonucleoprotein (RNP) and viral particles and is essential for RNA synthesis and virus replication (Sun et al., 2018). The M segment encodes the glycoprotein precursor (Gp), while the L segment encodes an RNA-dependent RNA polymerase (RdRp) (Ferron et al., 2017).

Both viruses were first isolated from *Phlebotomus chinensis* collected from the central region of China (Shanxi Province), with their geographical distribution now reported to be extensive (Wang et al., 2022). Currently, there have been no reports of disease caused by these two viruses. However, research indicates that WUXV can replicate and proliferate in various mammalian cell lines, inducing noticeable cytopathic effects (CPE) (Yao et al., 2022). Furthermore, WUXV has been reported to cause neurological symptoms and even death in mice (Wang et al., 2021). Researchers have also detected neutralizing antibodies in the sera of healthy individuals and chickens from the region where WUXV was isolated (Wang et al., 2021), suggesting that WUXV can infect both humans and chickens. In contrast, studies on HEDV are more limited and the virus has been found to replicate in mammalian cell (BHK-21 cells), however, since this virus does not cause CPE in BHK cells and viral plaques are not detectable in the cells (Xu et al., 2021), it is not possible to use plaque reduction neutralization tests to detect HEDV-neutralizing antibodies in human or animal serum samples. As a consequence, it is not possible to experimentally confirm whether or not HEDV infects humans and animals using these methods. As a result, the public health significance of the virus cannot be assessed within the context of virus gene molecular evolution, despite its placement on the same evolutionary branch as the medically significant Rift Valley Fever virus (RVFV) (Xu et al., 2021).

To investigate the infection status of HEDV and WUXV in humans and mammals from the regions where the viruses have been found, this study employed gene recombinant expression methods to obtain recombinant expressed proteins of the N genes of HEDV and WUXV. Protein-protein interactions (PPIs) were used to examine serum samples from febrile patients with an unidentified infection and local animals (i.e., dogs and chickens) from the aforementioned regions. The aim of the study was to investigate the infection situation of these two viruses in mammals and humans.

## 2 Materials and Methods

### 2.1 Induction of HEDV N protein expression

The sequence of the HEDV N protein was derived from the strain SXYQ1867-2 (GenBank: NC_079012). The 6×His tag was added to the N-terminal of the protein for purification. After codon optimization, the nucleotide sequence was ligated into the expression vector pCold TF (Tongyong Biotech, Anhui, China). The construct was transformed into *Escherichia coli* BL21(DE3) (Vazyme Biotech Co., Ltd, Nanjing, China), with single colonies then picked and inoculated into Luria-Bertani (LB) liquid medium containing 100 μg/ml ampicillin, followed by culture at 37°C for 16 h. When the OD_600_ reached 0.8, the culture was diluted 1:100 and transferred to fresh medium. After culturing at 37°C for 2-3 h, the cells were induced with 0.5 mmol/L isopropyl β-D-thiogalactopyranoside (IPTG) at 16°C for 18-20 h. The bacteria were harvested by centrifugation and resuspended in a buffer (500 mM NaCl, 20 mM Tris–HCl, pH 7.0), followed by disruption using an ultrasonic cell crusher (Ningbo Scientz Biotechnology Co., Ltd). The lysate was then centrifuged at 10,000 rpm for 10 min at 4°C to separate the supernatant and the pellet. The samples were supplemented with protein loading buffer, boiled, and centrifuged. Finally, SDS-PAGE was used to assess the protein expression levels (Li et al., 2022).

### 2.2 Purification and identification of HEDV N protein

Upon confirming proper protein expression, a large-scale induction was carried out. The soluble expression product was filtered through a 0.45 μm syringe filter and then purified using Ni-Sepharose chromatography (GE Healthcare, Chicago, USA). The purification efficiency was verified by SDS-PAGE and Western blot analysis. In addition, the amino acid sequence of the HEDV-N protein was determined based on LC-MS/MS to further validate the correctness of the protein. The purified protein was stored at -80°C after dialysis, while the WUXV N protein was maintained in our laboratory (Yao et al., 2022).

### 2.3 Homology analysis and tertiary structure

Because both WUXV and HEDV are viruses isolated from sandflies collected in Wuxiang County and belong to *Bunyaviruses*, the homology of the WUXV and HEDV N proteins was analyzed by Geneious software (Li et al., 2022), while the genetic evolution of RdRp was analyzed by MEGA5. An online software Swiss Model (https://swissmodel.expasy.org/interactive, accessed on June 10th, 2023) was used to analyze the details of the tertiary structure of the N proteins of HEDV and WUXV (Haseeb et al., 2023), with the structure and surface charge density compared and analyzed using VMD software.

### 2.4 Protein identification and the protein-protein interaction assay

The protein concentration of the purified soluble expression product was measured using the BCA Protein Assay Kit (Beyotime Biotechnology, Jiangsu, China). A 0.1 μg aliquot of the protein was separated on a 10% SDS-polyacrylamide gel and transferred onto a nitrocellulose membrane. The membrane was blocked for 2 h at room temperature (RT) in phosphate buffered saline containing Tween 20 (PBST) and 5% skim milk. The membrane was then incubated overnight at 4°C with the primary antibodies: Anti-his mouse monoclonal antibody (AC002, AB clonal Biotechnology, Wuhan, China) at a dilution of 1:2000 and serum at a dilution of 1:100 in antibody dilution buffer (BE6266, EASYBIO, Beijing, China) containing 0.5M NaCl. The membrane was then incubated with the secondary antibodies at RT for 1 h. The secondary antibodies included peroxidase-labeled goat anti-mouse IgG (Zsgb-Bio, Beijing, China) at a dilution of 1:5000, peroxidase-labeled goat anti-human IgG (Zsgb-Bio, Beijing, China) at a dilution of 1:6000, peroxidase-conjugated rabbit anti-dog IgG (A9042, Sigma Chemical Co., St. Louis, MO, USA) at a dilution of 1:40000, and peroxidase-labeled goat anti-chicken IgG (14-24-06, Kirkegaard & Perry Laboratories, USA) at a dilution of 1:40000. Finally, the membrane was incubated with enhanced chemiluminescence reagents (Tanon, Shanghai, Beijing) and imaged using an automated chemiluminescence imaging system (Amersham Imager 600; GE Healthcare, USA).

### 2.5 Collection of sera from febrile patients, dogs, and chickens

**Figure 1:**
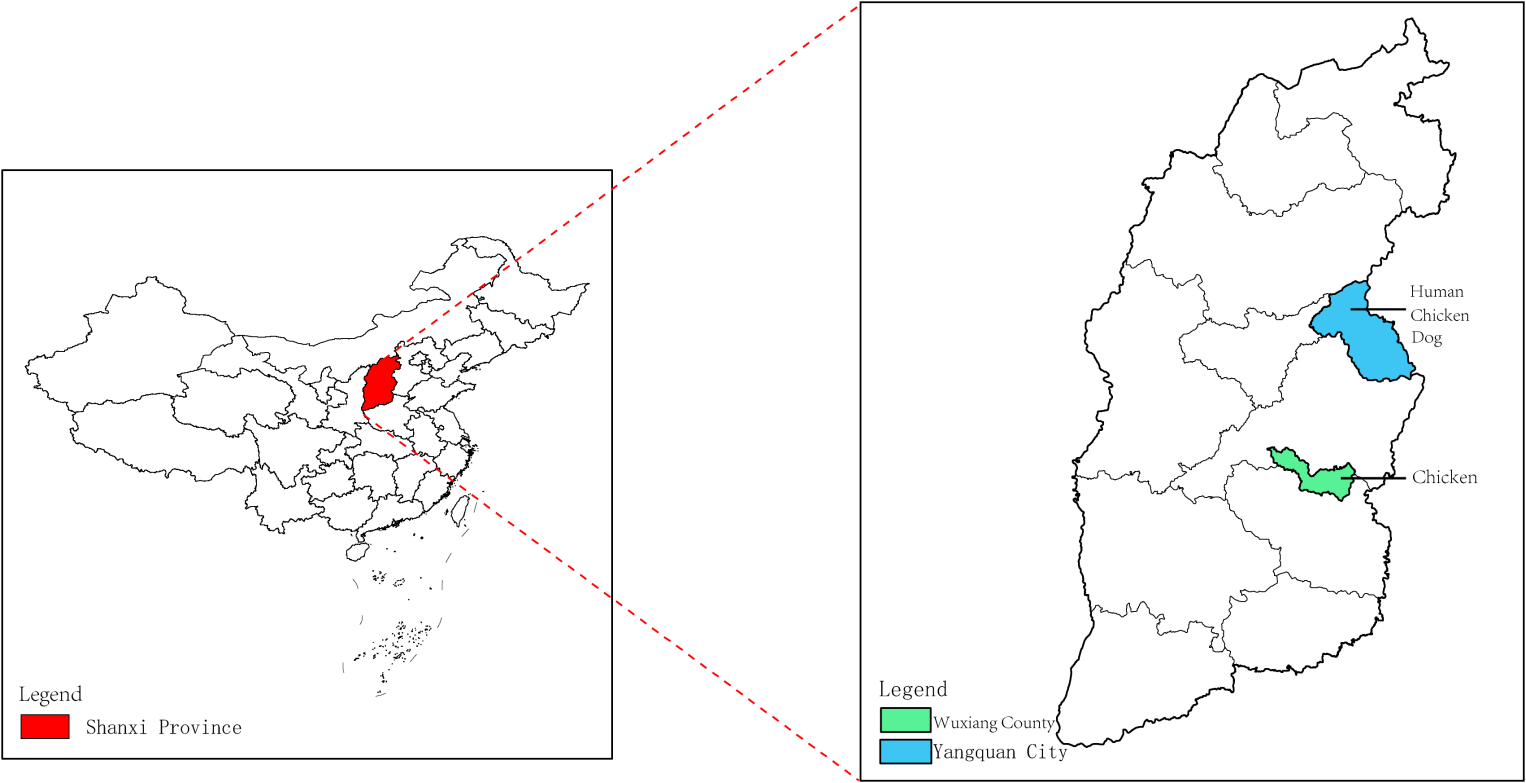
Collection of human, dog and chicken serum samples in the Shanxi Province. The red area in the figure represents the Shanxi Province in central China. The Wuxiang County (WX), where the specimens were collected for this study is shown by the green area, and the Yangquan County (YQ) by the blue area.

A total of 29 human serum samples were collected from patients in Yangquan City, Shanxi Province, China who had developed a fever of unknown cause between April 13, 2021, and August 26, 2021. In addition, 31 dog serum samples were obtained from a pet hospital in Yangquan City, Shanxi Province, while 36 chicken serum samples were collected, with 20 originating from Yangquan City, Shanxi Province in 2018, and 16 from the Wuxiang County in 2019 (**Supplementary Table 1**). The geographic distribution of the serum samples is illustrated in **Figure 1**. The serum samples were transported on ice to the laboratory and stored in a freezer at low temperatures for subsequent analysis.

## 3 Results

### 3.1 Expression, purification, and identification of recombinant HEDV-N protein

**Figure 2:**
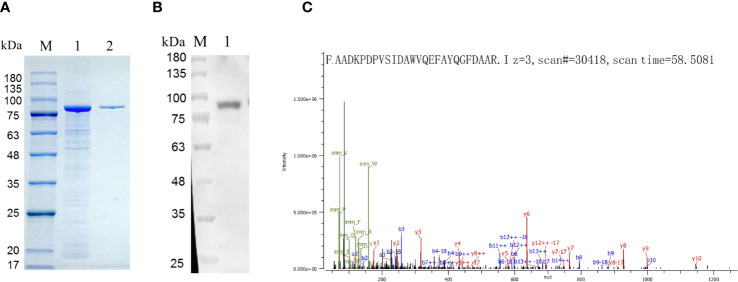
Identification of the HEDV N protein expressed in (*E*) *coli*. **(A)** SDS-PAGE showing expression of recombinant HEDV N protein in *E. coli*. M, protein marker; lane 1, supernatant of *E. coli* lysed cells; line 2, purified protein. **(B)** Western blot result of the recombinant HEDV N protein. M, protein marker; lane 1, purified HEDV N protein. **(C)** The amino acid sequence of HEDV-N protein was determined by protein N-terminal sequencing based on LC-MS/MS.

The expression of recombinant HEDV-N protein was successfully achieved in the lysates of *Escherichia coli* cells, as demonstrated by SDS-PAGE analysis. The predicted molecular weight of the N protein was 27 kDa. As shown in **Figure 2A**, the insertion of sequences such as the Trigger Factor tag (∼51 kDa) into the plasmid vector resulted in a recombinant N protein of approximately 78 kDa. Verification of HEDV-N protein fusion expression was confirmed using anti-His monoclonal antibodies in a Western blot analysis (**Figure 2B**). After purification using Ni^2+^-NTA agarose, the purity of the recombinant HEDV-N protein exceeded 90%. Furthermore, the N-terminal sequence of the protein, spanning amino acids 13 to 38, was determined by LC-MS/MS-based protein N-terminal sequencing, which showed that the region matched the expected sequence (**Figure 2C**; **Supplementary Table 2**).

### 3.2 Homology analysis and tertiary structure of WUXV-N and HEDV-N

**Figure 3:**
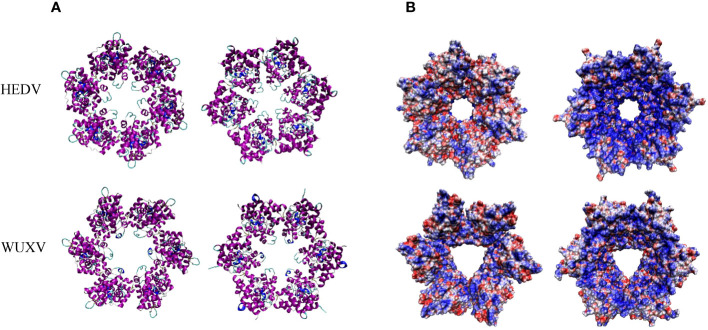
Tertiary structural and surface charge model of the HEDV and WUXV N proteins. **(A)** The tertiary structure of the HEDV and WUXV N proteins showed a positive bitmap of their surface charge models with their planes rotated by 180 degrees. **(B)** Blue represents a positive charge and red a negative charge.

The nucleotide homology between WUXV-N and HEDV-N was 52.33%, and the amino acid homology was 46.74%. There were some structural differences in the three-dimensional structure of HEDV and WUXV N proteins, and their surface charge distribution was obviously different. As shown in **Figure 3**, the negative charge area of HEDV was significantly higher than that of WUXV. The surface charge of protein is related to the interaction between protein-protein or protein-nucleic acid, the folding stability of proteins, and the dissolution and precipitation of proteins (Gitlin et al., 2006; Schroer et al., 2020).

### 3.3 Hybridization of the HEDV and WUXV N proteins with human and animal serum specimens

As shown in **Table 1**, of the 29 human sera, 17.24% (5/29) were positive for HEDV antibodies and 68.96% (20/29) were positive for WUXV antibodies. Analysis of the 31 dog sera showed that 87.10% (27/31) were positive for HEDV antibodies and 70.97% (22/31) were positive for WUXV antibodies, while of the 36 chicken sera, 47.22% (17/36) were positive for HEDV antibodies and 52.78% (19/36) were positive for WUXV antibodies. In the human serum samples, the HEDV positive rate was 20% (2/10) for females and 15.79% (3/19) for males. In patients with a fever, those younger than 60 yr had a positive rate of 7.69% (1/13) while those aged 60 yr or older had a positive rate of 25% (4/16). For WUXV, the positive rate was 70% (7/10) for females and 68.42% (13/19) for males, while in patients with a fever the positive rate was 69.23% (9/13) in patients younger than 60 yr and 68.75% (11/16) in those aged 60 yr or older. In the dog serum samples, the HEDV positive rate was 83.33% (10/12) for females and 89.47% (17/19) for males, while for WUXV, the positive rate was 83.33% (10/12) for females and 63.16% (12/19) for males. In the chicken serum samples, the positive rate for HEDV was 56.25% (9/16) in samples collected from Wuxiang County and 40% (8/20) for samples from Yangquan City. For WUXV, the positive rate was 75% (12/16) for samples from Wuxiang County and 40% (7/20) for samples from Yangquan City. These results showed that in human serum samples, the positive rate for WUXV was higher than that for HEDV, while in the chicken serum samples, the virus positive rate for both HEDV and WUXV was higher in Wuxiang County compared to that in Yangquan City (**Table 1**).

**Table 1:** Detection of anti-HEDV N protein and anti-WUXV N protein IgG.

			HEDV		WUXV	
				Total		Total
Human	Gender	Female	20% (2/10)	17.24% (5/29)	70% (7/10)	68.96%(20/29)
		Male	15.79% (3/19)		68.42% (13/19)	
	Age	<60	7.69% (1/13)		69.23% (9/13)	
		≥60	25% (4/16)		68.75% (11/16)	
Dog	Gender	Female	83.33% (10/12)	87.10% (27/31)	83.33% (10/12)	70.97%(22/31)
		Male	89.47% (17/19)		63.16% (12/19)	
Chicken	Area	Wuxiang county	56.25% (9/16)	47.22% (17/36)	75% (12/16)	52.78%(19/36)
					35% (7/20)	
		Yangquan county	40% (8/20)			
Total			51.04% (49/96)		63.54% (61/96)	

## 4 Discussion

Both HEDV and WUXV were first isolated from sandflies collected in the Shanxi Province in 2018 and belong to the *phlebovirus* genus. Studies have shown that WUXV is not an occasional virus in the Wuxiang County, but rather a stable viral population present in the local sandfly population (Xu et al., 2021). Furthermore, research has shown that HEDV and WUXV are not limited to the Wuxiang County but also exist in several other cities within the Shanxi Province. Meta-transcriptomics sequencing analysis in the previous study revealed a high prevalence and abundance of HEDV and WUXV in the investigated regions (Wang et al., 2022). However, their public health significance remains unclear, possibly due to these pathogens causing sporadic self-limiting diseases with non-specific symptoms such as fever, rash, and nausea. These symptoms are as mild as those caused by other *Phlebovirus* (Travassos et al., 1983) and *rickettsiae* (Jia et al., 2014; Jiao et al., 2022) infections, often termed self-healing diseases, with patients with the diseases less likely to be detected without active surveillance. At present, there are two methods to detect WUXV RNA (Hu et al., 2022; Yao et al., 2022), but given the transient presence of viral nucleic acids, detecting viral genes within patients presents challenges. In the current study we carried out viral gene amplification on specimens with positive hybridization results for HEDV and/or WUXV N proteins, although these experiments did not yield positive outcomes (results not shown). Generally, IgG antibodies appear 7-10 days after a viral infection and persist for several months or even a lifetime (Sun et al., 2023). As the N protein of *Bunyaviruses* serves is a key structural component of viral ribonucleoproteins (RNPs) and viral particles that participate directly in RNA synthesis and viral replication (Mo et al., 2020). And through the prediction of the tertiary structure of HEDV and WUXV, it was found that there are differences in their structure and surface charge, which may alter the RNA binding mode or binding efficiency. Therefore we chose to carry out serological testing of the HEDV and WUXV N proteins as antigens in our study.

Sandflies prefer to inhabit warmer and more humid environments and are often found near villages (Pareyn et al., 2019). Given their habitat within human settlements and among domesticated livestock (Guanghua et al., 1992) such as chickens, dogs, cows, pigs, and goats, studies on bloodmeal sources of sandflies collected from the Chinese Loess Plateau showed that chickens and humans were the most common sources of blood for sandflies in this region. Dogs, goats, cows, and pigs also serve as sources for blood in sandflies (Chen et al., 2020). In the Sichuan Jiuzhaigou region of China, pigs are the primary blood source for sandflies, followed by chickens and dogs (Chen et al., 2016). Sandflies also act as reservoir hosts for certain viruses like TOSV (Laroche et al., 2023), the sandfly fever Sicilian virus (SFSV) (Al-Numaani et al., 2023) and sandfly fever Naples virus (SFNV) (Reeves et al., 2015), in addition to being major vectors for Leishmania parasites (Volf et al., 2008). As blood-feeding insects, sandflies transmit viruses by biting humans and other mammals. Sandflies in the Wuxiang County exhibit a single generation growth pattern, with their appearance in May reaching a peak population between June and July, before disappearing in September (Xiaodong et al., 2023). This study detected positive WUXV antibody responses in the sera of febrile patients with an unidentified infection. Our analyses showed that the positive rate of WUXV antibodies in sera collected was 40% (2/5) in April, 60% (3/5) in May, 50% (4/8) in June, 100% (3/3) in July, and 100% (8/8) in August. The positive rate of WUXV antibodies in the sera of febrile patients with an unidentified infection was also shown to correlate with the density of sandflies. Unfortunately, due to a lack of detailed information on the month of collection for the dog and chicken serum samples, a similar analysis could not be conducted for these animals, and represented a limitation of our study. Moreover, in chickens, the positive rates for both viruses were higher in Wuxiang County than in Yangquan City, due potentially to geographic and environmental differences between the two areas, as well as variations in the practices used for poultry farming.

Molecular genetic evolution analysis based on the RdRp of the viruses showed that the WUXV clustered with Toros viruses (Alkan et al., 2016) and Corfou virus (Calzolari et al., 2021), while HEDV clustered with the RVFV (Connors and Hartman, 2022), forming a specific shared evolutionary branch with that virus (**Supplementary Figure 1**). These two viruses appear to be distinct isolates of the same viral species. Notably, HEDV is the closest relative to the RVFV discovered to date. Studies using diagnostic tests with RVFV antigens have also suggested the potential for cross-reactivity of antibodies induced by viruses belonging to a sandfly-transmitted virus genus (Wu et al., 2014). While cross-reactivity in serological tests of *Phlebovirus* is a recognized phenomenon, our laboratory does not have a RVFV strain that prevented us from conducting cross-neutralization tests to understand the extent of antigenic cross-reactivity between the two viruses. Therefore, a further limitation of our study was that we were unable to infer a relationship between the HEDV-positive specimens and the RVFV.

While our findings demonstrate the presence of HEDV and WUXV infections among local febrile patients, it remains uncertain whether the patients’ fevers were attributable to a sandfly-borne virus infection. Therefore, it is necessary to detect differences in the antibodies against HEDV and WUXV in acute and convalescent serum samples, as well as IgM antibodies against the virus produced in the acute phase and IgG antibodies produced during the convalescent phase in patients. This would allow the relationship between these antibodies and infectious diseases in the local population to be determined. Furthermore, the isolation of multiple virus strains from sandflies collected in chicken coops and sheep pens in Wuxiang County suggests that chickens and sheep may be important hosts for WUXV in the area (Wang et al., 2021). Although specific antibodies to HEDV and WUXV were detected in chicken and dog sera, the lack of data regarding the health status of these animals precludes determining whether HEDV and WUXV are capable of causing diseases in domesticated animals. In addition, due to the limited sample size and absence of temporal continuity, a comprehensive and systematic understanding of HEDV and WUXV infections among humans and animals in the Shanxi Province could not be obtained. Furthermore, serological study lacks some control, the serum from non-endemic regions will be considered in subsequent ongoing surveillance to further confirm the positive results are truly associated with past infection of the two viruses. Further validation through the collection of a larger number of samples is therefore warranted to address these limitations.

In summary, our study demonstrated the presence of HEDV and WUXV infections among humans, dogs, and chickens in Yangquan City and the Wuxiang County. The infection rate of HEDV in animals (dogs and chickens) was 65.67% (44/67), significantly higher than the human infection rate of 17.24% (5/29). However, the infection rate of WUXV in animals (dogs and chickens) was 61.19% (41/67), comparable to that observed for the human infection rate of 68.96% (20/29). We also conducted specific antibody testing for HEDV and WUXV in human and animal serum samples from the Shanxi Province. This was the first investigation on the infection status of these newly isolated sandfly-borne viruses in mammals and their relationship with diseases. As sandfly-borne viruses can cause diseases in both humans and animals and are zoonotic pathogens, although we could not definitively establish whether HEDV and WUXV were responsible for specific illnesses such as fever, continued surveillance of their prevalence in the area is essential. Strengthening the detection and monitoring of sandfly-transmitted viruses causing diseases in humans and animals holds significant public health importance for China and even East Asia.

## Data availability statement

The original contributions presented in the study are included in the article/**Supplementary Material**. Further inquiries can be directed to the corresponding authors.

## Ethics statement

The studies involving humans were approved by National Institute for Viral Disease Control and Prevention, China CDC. The studies were conducted in accordance with the local legislation and institutional requirements. The human samples used in this study were acquired from primarily isolated as part of your previous study for which ethical approval was obtained. Written informed consent for participation was not required from the participants or the participants’ legal guardians/next of kin in accordance with the national legislation and institutional requirements. The animal study was approved by National Institute for Viral Disease Control and Prevention, China CDC. The study was conducted in accordance with the local legislation and institutional requirements.

## Author contributions

XY: Methodology, Writing – original draft. QY: Data curation, Writing – review & editing. XT: Resources, Writing – review & editing. YKZ: Software, Writing – review & editing. HL: Resources, Writing – review & editing. SF: Investigation, Writing – review & editing. ZL: Methodology, Writing – review & editing. YJZ: Methodology, Writing – review & editing. FL: Investigation, Writing – review & editing. WZ: Supervision, Writing – review & editing. YH: Investigation, Writing – review & editing. RW: Software, Writing – review & editing. BW: Resources, Writing – review & editing. KN: Funding acquisition, Project administration, Writing – review & editing. SX: Funding acquisition, Writing – review & editing. JC: Resources, Writing – review & editing. XL: Supervision, Writing – review & editing. HW: Funding acquisition, Supervision, Writing – review & editing. GL: Resources, Supervision, Writing – review & editing.

## References

[B1] Alkan C. Erisoz K. O. Alten B. de Lamballerie X. Charrel R. N. (2016). Sandfly-Borne Phlebovirus Isolations from Turkey: New Insight into the Sandfly fever Sicilian and Sandfly fever Naples Species. PloS Negl. Trop. Dis. 10 (3), e4519. doi: 10.1371/journal.pntd.0004519 PMC4805245 27007326

[B2] Al-Numaani S. A. Al-Nemari A. T. El-Kafrawy S. A. Hassan A. M. Tolah A. M. Alghanmi M.. (2023). Seroprevalence of Toscana and sandfly fever Sicilian viruses in humans and livestock animals from western Saudi Arabia. One Health 17, 100601. doi: 10.1016/j.onehlt.2023.100601 37520847 PMC10372353

[B3] Calzolari M. Romeo G. Callegari E. Bonilauri P. Chiapponi C. Carra E.. (2021). Co-circulation of phleboviruses and leishmania parasites in sand flies from a single site in Italy monitored betwee 2017 and 2020. Viruses 13 (8), 1660. doi: 10.3390/v13081660 34452524 PMC8402820

[B4] Calisher C. H. Calzolari M. (2021). Taxonomy of phleboviruses, emphasizing those that are sandfly-borne. Viruses 13 (5), 918. doi: 10.3390/v13050918 34063467 PMC8156068

[B5] Chen H. M. Chen H. Y. Tao F. Gao J. P. Li K. L. Shi H.. (2020). Leishmania infection and blood sources analysis in Phlebotomus chinensis (Diptera: Psychodidae) along extension region of the loess plateau, China. Infect. Dis. Poverty 9 (1), 125. doi: 10.1186/s40249-020-00746-8 32867841 PMC7461359

[B6] Chen H. Li K. Shi H. Zhang Y. Ha Y. Wang Y.. (2016). Ecological niches and blood sources of sand fly in an endemic focus of visceral leishmaniasis in Jiuzhaigou, Sichuan, China. Infect. Dis. Poverty 5 (1), 33. doi: 10.1186/s40249-016-0126-9 27075573 PMC4831150

[B7] Connors K. A. Hartman A. L. (2022). Advances in understanding neuropathogenesis of rift valley fever virus. Annu. Rev. Virol. 9 (1), 437–450. doi: 10.1146/annurev-virology-091919-065806 36173701 PMC10316117

[B8] Ferron F. Weber F. de la Torre J. C. Reguera J. (2017). Transcription and replication mechanisms of Bunyaviridae and Arenaviridae L proteins. Virus Res. 234, 118–134. doi: 10.1016/j.virusres.2017.01.018 28137457 PMC7114536

[B9] Gitlin I. Carbeck J. D. Whitesides G. M. (2006). Why are proteins charged? Networks of charge-charge interactions in proteins measured by charge ladders and capillary electrophoresis. Angew Chem. Int. Ed Engl. 45 (19), 3022–3060. doi: 10.1002/anie.200502530 16619322

[B10] Haseeb M. Amir A. Ikram A. (2023). In silico analysis of SARS-coV-2 spike proteins of different field variants. Vaccines (Basel) 11 (4), 736. doi: 10.3390/vaccines11040736 37112648 PMC10145761

[B11] Hu D. Yao X. Fu S. Li F. Gu T. Chen J.. (2022). Establishment of TaqMan RT-PCR assay for Wuxiang virus. Chin. J. Exp. Clin. Virol. 36 (4), 460–464. doi: 10.3760/cma.j.cn112866-20220310-00056

[B12] Jia N. Zheng Y. C. Ma L. Huo Q. B. Ni X. B. Jiang B. G.. (2014). Human infections with Rickettsia raoultii, China. Emerg. Infect. Dis. 20 (5), 866–868. doi: 10.3201/eid2005.130995 24750663 PMC4012798

[B13] Jiao J. Yu Y. He P. Wan W. Yang X. Wen B.. (2022). First detection of Rickettsia aeschlimannii in Hyalomma marginatum in Tibet, China. Zoonoses 2 (1). doi: 10.15212/ZOONOSES-2022-0026

[B14] Laroche L. Ayhan N. Charrel R. Bañuls A. L. Prudhomme J. (2023). Persistence of Toscana virus in sugar and blood meals of phlebotomine sand flies: epidemiological and experimental consequences. Sci. Rep. 13 (1), 5608. doi: 10.1038/s41598-023-32431-9 37019992 PMC10076283

[B15] Li D. Zhang Q. Liu Y. Wang M. Zhang L. Han L.. (2022). Indirect ELISA Using Multi-Antigenic Dominants of p30, p54 and p72 Recombinant Proteins to Detect Antibodies against African Swine Fever Virus in Pigs. Viruses 14 (12), 2660. doi: 10.3390/v14122660 36560663 PMC9782230

[B16] Mo Q. Xu Z. Deng F. Wang H. Ning Y. J. (2020). Host restriction of emerging high-pathogenic bunyaviruses via MOV10 by targeting viral nucleoprotein and blocking ribonucleoprotein assembly. PloS Pathog. 16 (12), e1009129. doi: 10.1371/journal.ppat.1009129 33284835 PMC7746268

[B17] Pareyn M. Van den Bosch E. Girma N. van Houtte N. Van Dongen S. Van der Auwera G.. (2019). Ecology and seasonality of sandflies and potential reservoirs of cutaneous leishmaniasis in Ochollo, a hotspot in southern Ethiopia. PloS Negl. Trop. Dis. 13 (8), e7667. doi: 10.1371/journal.pntd.0007667 PMC6715250 31425506

[B18] Reeves W. K. Szymczak M. S. Burkhalter K. L. Miller M. M. (2015). Laboratory validation of the sand fly fever virus antigen assay. J. Am. Mosq. Control Assoc. 31 (4), 380–383. doi: 10.2987/moco-31-04-380-383.1 26675463 PMC7111559

[B19] Schroer C. Baldauf L. van Buren L. Wassenaar T. A. Melo M. N. Koenderink G. H.. (2020). Charge-dependent interactions of monomeric and filamentous actin with lipid bilayers. Proc. Natl. Acad. Sci. U.S.A. 117 (11), 5861–5872. doi: 10.1073/pnas.1914884117 32123101 PMC7084070

[B20] Sun J. Yang Z. D. Xie X. Li L. Zeng H. S. Gong B.. (2023). Clinical application of SARS-CoV-2 antibody detection and monoclonal antibody therapies against COVID-19. World J. Clin. cases 11 (10), 2168–2180. doi: 10.12998/wjcc.v11.i10.2168 37122515 PMC10131020

[B21] Sun Y. Li J. Gao G. F. Tien P. Liu W. (2018). Bunyavirales ribonucleoproteins: the viral replication and transcription machinery. Crit. Rev. Microbiol. 44 (5), 522–540. doi: 10.1080/1040841X.2018.1446901 29516765

[B22] Ter Horst S. Conceicao-Neto N. Neyts J. Rocha-Pereira J. (2019). Structural and functional similarities in bunyaviruses: Perspectives for pan-bunya antivirals. Rev. Med. Virol. 29 (3), e2039. doi: 10.1002/rmv.2039 30746831 PMC7169261

[B23] Travassos D. R. A. Tesh R. B. Pinheiro F. P. Travassos da Rosa J. F. Peterson N. E. (1983). Characterization of eight new phlebotomus fever serogroup arboviruses (Bunyaviridae: Phlebovirus) from the Amazon region of Brazil. Am. J. Trop. Med. Hyg 32 (5), 1164–1171. doi: 10.4269/ajtmh.1983.32.1164 6312820

[B24] Volf P. Hostomska J. Rohousova I. (2008). Molecular crosstalks in Leishmania-sandfly-host relationships. Parasite 15 (3), 237–243. doi: 10.1051/parasite/2008153237 18814687

[B25] Wang J. Fan N. Fu S. Cheng J. Wu B. Xu Z.. (2021). Isolation and characterization of wuxiang virus from sandflies collected in yangquan county, shanxi province, China. Vector Borne Zoonotic Dis. 21 (6), 446–457. doi: 10.1089/vbz.2020.2699 33891486

[B26] Wang Q. Fu S. Cheng J. Xu X. Wang J. Wu B.. (2021). Re-isolation of wuxiang virus from wild sandflies collected from yangquan county, China. Virol. Sin. 36 (5), 1177–1186. doi: 10.1007/s12250-021-00398-4 34057679 PMC8165349

[B27] Wang J. Fu S. Xu Z. Cheng J. Shi M. Fan N.. (2020). Emerging sand fly-borne phlebovirus in China. Emerg. Infect. Dis. 26 (10), 2435–2438. doi: 10.3201/eid2610.191374 32946723 PMC7510709

[B28] Wang J. Gou Q. Y. Luo G. Y. Hou X. Liang G. Shi M. (2022). Total RNA sequencing ofPhlebotomus chinensis sandflies in China revealed viral, bacterial, and eukaryotic microbes potentially pathogenic to humans. Emerging Microbes Infections 11 (1), 2080–2092. doi: 10.1080/22221751.2022.2109516 35916448 PMC9448391

[B29] Wang Q. Yin Q. Fu S. Cheng J. Xu X. Wang J.. (2022). Isolation and identification of sandfly-borne viruses from sandflies collected from june to august, 2019, in yangquan county, China. Viruses 14 (12), 2692. doi: 10.3390/v14122692 36560697 PMC9782482

[B30] Wu W. Zhang S. Zhang Q. F. Li C. Liang M. F. Li D. X. (2014). Study on serological cross-reactivity of six pathogenic phleboviruses. Bing Du Xue Bao 30 (4), 387–390. 25272591

[B31] Tian X. Dai P. Zhao J. Dong H. Cheng J. (2023). Distribution and seasonal fluctuation of sandflies in Shanxi province,China. Chin. J. Vector Biol. Control 34 (3), 417–421. doi: 10.11853/j.issn.1003.8280.2023.03.023

[B32] Xiong G. Jin C. Chen X. Hong Y. Su Z. Liu P. (1992). Study on the Biology of Sandfly in Longnan and Northern Sichuan and its Relationship with Visceral leishmaniasis in Human and Dogs. WUYI Sci. J. 1, 7–18.

[B33] Xu X. Cheng J. Fu S. Wang Q. Wang J. Lu X.. (2021). Wuxiang virus is a virus circulated naturally in wuxiang county, China. Vector Borne Zoonotic Dis. 21 (4), 289–300. doi: 10.1089/vbz.2020.2702 33600240

[B34] Xu Z. Fan N. Hou X. Wang J. Fu S. Song J.. (2021). Isolation and identification of a novel phlebovirus, hedi virus, from sandflies collected in China. Viruses 13 (5), 772. doi: 10.3390/v13050772 33925561 PMC8145316

[B35] Yao X. H. Hu D. H. Fu S. H. Li F. He Y. Yin J. Y.. (2022). A reverse-transcription recombinase-aided amplification assay for the rapid detection of the wuxiang virus. BioMed. Environ. Sci. 35 (8), 746–749. doi: 10.3967/bes2022.096 36127786 PMC10089701

[B36] Yao X. Yin Q. Hu D. Fu S. Zhang W. Nie K.. (2022). *In vitro* infection dynamics of wuxiang virus in different cell lines. Viruses 14 (11), 2383. doi: 10.3390/v14112383 36366481 PMC9699334

