# Supplementary figures and images for: Human and animal exposure to newly discovered sand fly viruses, China
Source: Front Cell Infect Microbiol. 2024 Jan 3;13:1291937. doi: 10.3389/fcimb.2023.1291937 (PMC10791927; doi:10.3389/fcimb.2023.1291937)

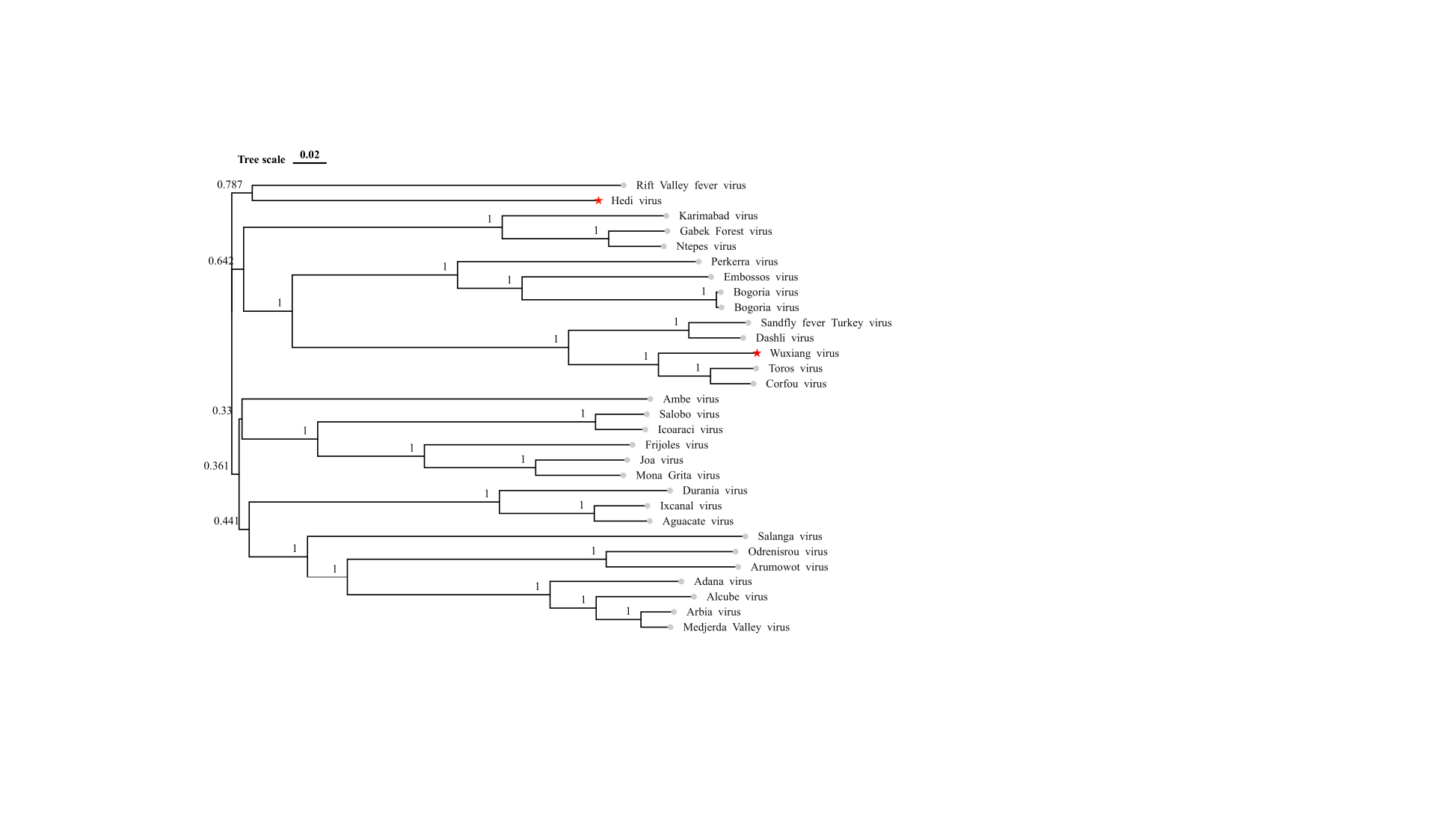

Supplement: Supplementary file 3 [file Image_1.tif]
